# Associations between White Matter Microstructure and Cognitive Performance in Old and Very Old Age

**DOI:** 10.1371/journal.pone.0081419

**Published:** 2013-11-25

**Authors:** Erika J. Laukka, Martin Lövdén, Grégoria Kalpouzos, Tie-Qiang Li, Tomas Jonsson, Lars-Olof Wahlund, Laura Fratiglioni, Lars Bäckman

**Affiliations:** 1 Aging Research Center, Karolinska Institutet and Stockholm University, Stockholm, Sweden; 2 Department of Medical Physics, Karolinska Institutet, Stockholm, Sweden; 3 Department of Clinical Geriatrics, Karolinska Institutet, Stockholm, Sweden; 4 Stockholm Gerontology Research Center, Stockholm, Sweden; University Of Cambridge, United Kingdom

## Abstract

Increasing age is associated with deficits in a wide range of cognitive domains as well as with structural brain changes. Recent studies using diffusion tensor imaging (DTI) have shown that microstructural integrity of white matter is associated with cognitive performance in elderly persons, especially on tests that rely on perceptual speed. We used structural equation modeling to investigate associations between white matter microstructure and cognitive functions in a population-based sample of elderly persons (age ≥ 60 years), free of dementia, stroke, and neurological disorders (n = 253). Participants underwent a magnetic resonance imaging scan, from which mean fractional anisotropy (FA) and mean diffusivity (MD) of seven white matter tracts were quantified. Cognitive functioning was analyzed according to performance in five task domains (perceptual speed, episodic memory, semantic memory, letter fluency, and category fluency). After controlling for age, FA and MD were exclusively related to perceptual speed. When further stratifying the sample into two age groups, the associations were reliable in the old-old (≥78 years) only. This relationship between white matter microstructure and perceptual speed remained significant after excluding persons in a preclinical dementia phase. The observed pattern of results suggests that microstructural white matter integrity may be especially important to perceptual speed among very old adults.

## Introduction

Diffusion tensor imaging (DTI) is a non-invasive magnetic resonance imaging (MRI) technique that is sensitive to the microstructure of white matter. DTI measures the diffusion of water molecules in brain tissue and enables visualization of white matter pathways and quantification of aspects of microstructure, with measures such as fractional anisotropy (FA) and mean diffusivity (MD). Higher FA is associated with greater directionality of water molecules and implies higher fiber density or coherence in a voxel. In contrast, higher MD reflects higher rate of diffusion and implies less dense tissue. Aging is typically associated with decreases in FA and increases in MD, probably resulting from, for example, axonal degeneration and loss of myelin [1]. Normal aging is also associated with deficits in many cognitive domains, with the largest age-related differences usually observed for perceptual speed [2,3]. In recent years, several studies have examined associations between indices of white matter microstructure and cognitive performance. Lower FA and higher MD in normal-appearing white matter in elderly persons has most consistently been associated with worse performance on tests tapping perceptual speed and executive functioning [4-6]. 

Given the association between white matter connections and processing speed, both known to deteriorate in aging, it has been suggested that decline in white matter integrity could be an important biological correlate of age-related cognitive deficits, particularly for perceptual speed [7,8]. However, chronological age shares a substantial portion of the variance associated with the relation between white matter and cognition. Thus, there is still uncertainty about the extent to which age-related changes in cognitive performance are related to age-related changes in white matter structure, and to what extent these processes occur in parallel [9]. Few studies have focused on the white matter-cognition association in very old age (> 75 years), or examined the strength of the association in different age groups. Increases of the strength of the association in older age groups would be expected if changes in white matter integrity and cognitive performance are related [10-12], or if white matter integrity only impacts functioning after declining below a critical threshold. Other limitations in previous studies include small sample sizes and a narrow selection of cognitive tests.

We have previously demonstrated age-related differences in white matter microstructure and cognitive performance, using data from the Swedish National Study on Aging and Care in Kungsholmen (SNAC-K). Seven latent factors of white matter tracts, derived by structural equation modeling (SEM), all showed decreased FA and increased MD with increasing age [12]. Similarly, five latent cognitive abilities all showed negative correlations with age [13]. An advantage with performing analyses at the latent level is that it minimizes the effects of measurement error. Furthermore, observed associations are less dependent on task-specific influences.

For the present study, we had access to a large population-based sample of non-demented older adults (n = 253), who had taken part in a DTI scan and extensive cognitive testing. We aimed to examine possible associations between microstructural white matter integrity and cognitive performance at the latent level. A second aim was to examine the strength of these associations in different age segments, stratifying the sample into young-old (60-72 years) and old-old (78-87 years) age groups. Finally, we examined whether potential associations remained after excluding future dementia cases from the sample.

## Methods

### Participants

Between 2001 and 2004, 3363 persons, resident in the Kungsholmen area in central Stockholm, Sweden, participated in the baseline assessment of SNAC-K, a population-based study of persons aged ≥ 60 years. Participants were randomly selected based on their date of birth and belonged to pre-specified age cohorts (60, 66, 72, 78, 81, 84, 87, 90, 93, 96 years, and 99 years and older). The examination consisted of a nurse interview, a medical examination, and neuropsychological testing. In addition, a random subsample was asked to take part in an MRI examination. The effective sample used in this study (n = 253) included participants with acceptable quality of the DTI images. The bulk of participants (90%) were right-handed, 2% were left-handed, and 8% were ambiguous. The sample was screened for dementia, Parkinson’s disease, epilepsy, stroke, schizophrenia, and bipolar disorder. Because very few participants were older than 87 years, we excluded these subjects in order to have more homogenous age groups. An additional seven subjects were excluded because they did not participate in the cognitive testing. Selectivity with regard to educational background and cognitive performance of the effective sample for DTI analyses in relation to the total non-demented sample in SNAC-K was negligible [12]. The SNAC-K project has been approved by the ethical committee at Karolinska Institutet, Stockholm, Sweden, and the research was conducted according to the ethical guidelines expressed in the Declaration of Helsinki. Written informed consent was obtained from all participants.

### MRI acquisition

All MRI measurements were conducted using a 1.5T scanner (Philips Intera, Netherlands). DTI data were acquired using a single-shot diffusion-weighted echoplanar imaging sequence with the following parameters: FOV=230 × 138 mm^2^; 128 x 77 matrix; TE = 104 ms; TR = 6838 ms; slice thickness=5 mm with 1 mm gap; b-value 600 s/mm^2^. For all participants, a DTI scheme with 6 non-collinear diffusion-weighting gradient directions was used to determine the diffusion tensor set. 

A detailed description of how the DTI data were preprocessed and how FA and MD were derived has been provided elsewhere [12]. In short, after diffusion tensor calculation, these parameters were derived on a voxel-by-voxel basis using three steps: (1) estimation of eigenvalues and eigenvectors of the diffusion tensor using the single-value decomposition algorithm; (2) calculation of MD as the mean of the diagonal elements; and (3) calculation of FA according to its definition [14]. The FA data were further processed using tract-based spatial statistics (TBSS) [15] in FSL [16]. Here, the mean FA image was thinned to create a mean FA skeleton, which represents the centerlines of all tracts common to the sample. The mean skeleton was thresholded and binarized at FA > 0.2 to reduce the likelihood of partial voluming. Each participant’s aligned FA data were then projected onto this skeleton, resulting in individual skeleton images. Finally, the MD images were processed based on the results of the processing of the FA images. 

### White matter SEM model

**Figure 1:**
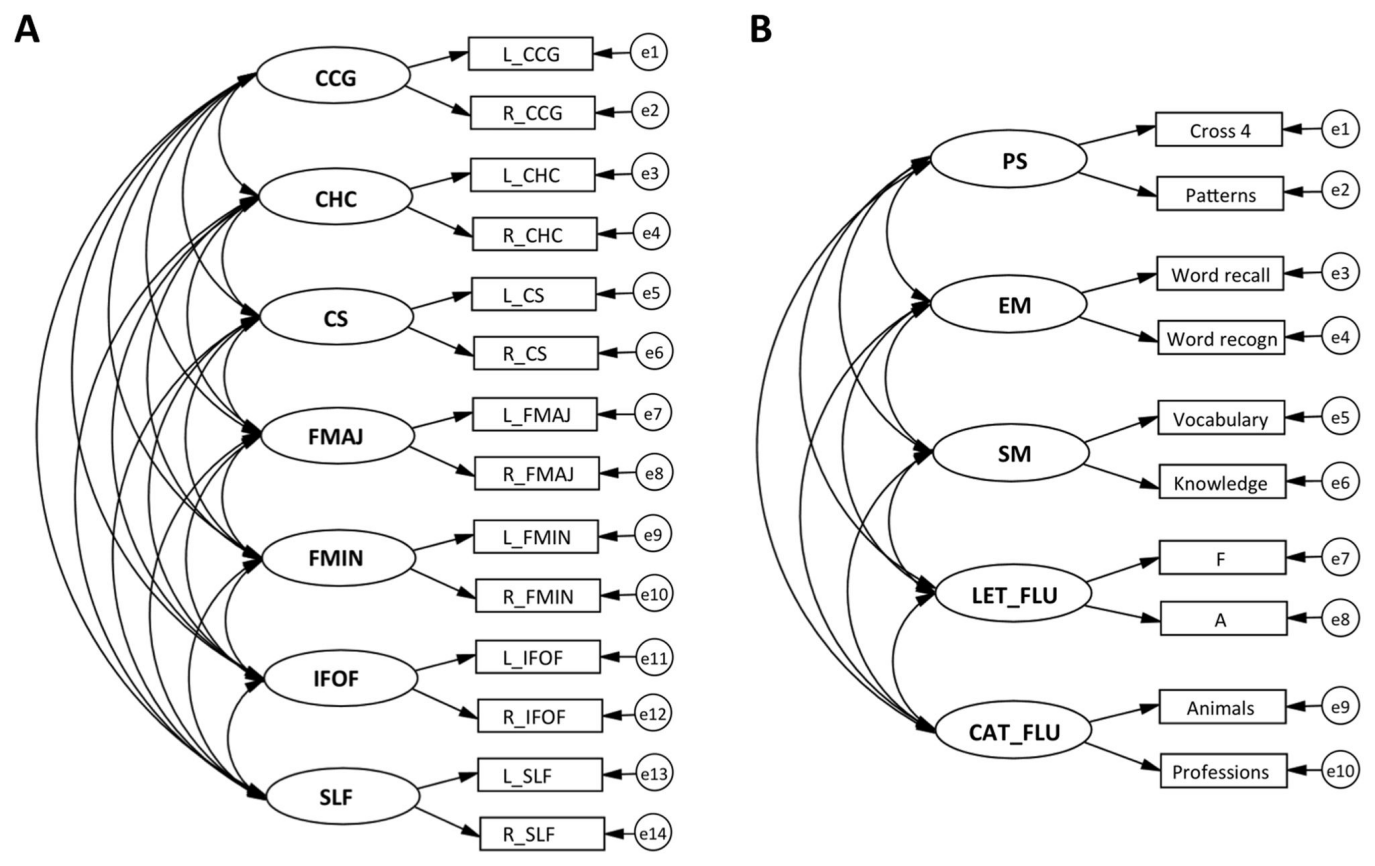
SEM models for the seven latent white matter factors (A) and the five latent cognitive factors (B). Latent factors are depicted with circles, observed variables with rectangles, regressions with one-headed arrows, and covariances with two-headed arrows. FA = fractional anisotropy, MD = mean diffusivity, CCG = cingulum cingulate gyrus, CHC = cingulum hippocampus, CS = corticospinal tract, FMAJ = forceps major, FMIN = forceps minor, IFOF = inferior fronto-occipital Fasciculus, SLF = superior longitudinal Fasciculus, PS = perceptual speed, EM = episodic memory, SM = semantic memory, LET_FLU = letter fluency, CAT_FLU = category fluency, L = left, R = right, e = error. Model fit for the combined models in the total sample: FA: χ^2^ = 241.31, df = 186, CFI = 0.99, RMSEA = 0.03; MD: χ^2^ = 325.01, df = 186, n = 253, CFI = 0.97, RMSEA = 0.05.

To prepare the data for SEM, we produced masks of seven tracts of interest in each hemisphere (see Figure 1A), with the procedures described and validated by Lövdén et al. [12]. The masks were: the cingulate gyrus part of cingulum (CCG), the portion of cingulum that extends to the hippocampus (CHC), the corticospinal tract (CS), the forceps major (FMAJ), the forceps minor (FMIN), the inferior fronto-occipital fasciculus (IFOF), and the superior longitudinal fasciculus (SLF). These 14 masks (7 tracts x 2 hemispheres) were used to extract mean FA and MD data from each individual’s skeleton image. The majority of the masks were based on the JHU white-matter tractography atlas [17,18], whereas the CS mask emanated from the Catani tractography atlas [19,20]. Separate SEMs were created for FA and MD. The latent factors were always formed by the left and right versions of each tract. Thus, the latent factors represent the common variance across hemispheres for a given tract. This approach was based on previous observations of high correlations between left and right white matter indicators in homologous tracts [12,21] and the validity of such a model was supported in previous work [12]. Model fit was evaluated with the Comparative Fit Index (CFI) and the Root-Mean-Square Error of Approximation (RMSEA). Acceptable model fit was defined as a CFI above 0.95 and an RMSEA below 0.08 [22]. We obtained good fit of a model positing that individual differences in white matter microstructure are organized according to tracts, both for FA (χ^2^ = 131.28, df = 126, *n* = 260, CFI = 0.998, RMSEA = 0.013) and MD (χ^2^ = 277.49, df = 126, *n* = 260, CFI = 0.953, RMSEA = 0.068) [12].

### Cognitive SEM model

For the cognitive data, we estimated a SEM with five latent cognitive factors (see Figure 1B): perceptual speed (PS), episodic memory (EM), semantic memory (SM), letter fluency (LET_FLU), and category fluency (CAT_FLU). The model was based on the following cognitive tests: PS – digit cancellation [23] and pattern comparison [24], EM – free recall of random words and word recognition [13], SM – vocabulary [25] and general knowledge [26], LET_FLU – words beginning with F and A [27], and CAT_FLU – animal and profession fluency [27]. For a full description of the cognitive tasks, the reader may consult Laukka et al. [13]. All models were estimated with full maximum likelihood, where information in the complete data set was used for estimating parameters that involved missing values. The model showed good fit (χ^2^= 76.72, df = 25, n = 2694, CFI = 0.995, RMSEA = 0.028), indicating that our categorization of the cognitive tests constitutes an adequate representation of the data [13].

### Statistical analyses

All analyses were performed with AMOS 5.0 (IBM SPSS 20). First, we assured good model fit for a model combining the white matter and cognitive SEM models described above: FA (χ^2^ = 241.31, df = 186, CFI = 0.99, RMSEA = 0.03) and MD (χ^2^ = 325.01, df = 186, n = 253, CFI = 0.97, RMSEA = 0.05). Standardized loadings on the latent factors and correlations among the latent white matter factors and the latent cognitive factors, respectively, are available as supporting information (Tables S1-S3 in File S1). Next, we examined the associations between the tract factors and the cognitive factors. All analyses were performed in the total sample as well as in two separate age groups, one young-old (60-72 years) and one old-old (78-87 years) group. Chronological age was included as a predictor of the latent white matter factors and the latent cognitive factors. Thus, the effect of age was covaried out in all analyses. We estimated covariances (and computed the correlations) between the residual terms (i.e., the residuals after accounting for the influence of chronological age) for all latent factors in the model. The significance testing of the correlation coefficients was based on the covariances and their associated standard errors. The threshold for statistical significance was set to p < 0.05. As a final step, we examined the associations between white matter microstructure and cognition in a sample where preclinical dementia cases had been excluded.

## Results

Background information for the total sample and for the two age groups is shown in Table 1. The mean age of the sample was 72.29 years and the majority of the participants (64.43%) were female. Note that educational level was relatively high and that participants were cognitively intact, as indicated by their MMSE scores. All variables analyzed in the combined SEM model displayed acceptable skewness and kurtosis [22], except for vocabulary (kurtosis = 2.52 in the total sample, 5.31 in the young-old, and 0.90 in the old-old). 

**Table 1:** Background information for the total sample and for the two age groups.

	Total sample		60-72 years		78-87 years	
	n = 253		n = 147		n = 106	
	M	SD	M	SD	M	SD
Age	72.29	8.88	65.64	4.78	81.51***	3.11
% female	64.43		63.27		66.04	
Education, years	12.22	3.96	12.97	3.64	11.17***	4.15
MMSE	29.10	1.01	29.32	0.85	28.80***	1.15
FA (mean of left+right)						
CCG	40.21	2.86	41.36	2.57	38.61***	2.46
CHC	39.83	2.66	40.74	2.56	38.58***	2.26
CS	56.14	2.29	56.80	2.21	55.21***	2.09
FMAJ	57.30	2.94	58.33	2.55	55.88***	2.86
FMIN	51.32	3.31	52.77	2.61	49.32***	3.13
IFOF	46.49	2.43	47.49	2.11	45.10***	2.15
SLF	41.70	2.43	42.34	2.23	40.79***	2.41
MD (mean of left+right)						
CCG	82.52	4.31	80.51	3.35	85.31***	3.93
CHC	100.36	9.31	95.91	7.38	106.53***	8.13
CS	75.09	2.73	73.79	1.76	76.89***	2.82
FMAJ	78.54	5.53	75.96	3.64	82.12***	5.73
FMIN	81.86	5.72	79.18	4.31	85.57***	5.37
IFOF	84.51	4.63	82.33	3.25	87.53***	4.57
SLF	77.58	4.32	75.68	3.09	80.23***	4.41
PS						
Digit cancellation	18.18	4.19	19.53	4.21	16.27***	3.35
Pattern comparison	14.68	3.72	16.33	3.28	12.34***	2.98
EM						
Word recall	7.17	2.41	7.80	2.27	6.29***	2.33
Word recognition	6.89	4.28	7.50	4.34	6.04**	4.07
(remember)						
SM						
Vocabulary	23.64	4.38	24.55	3.81	22.39***	4.81
General knowledge	7.06	1.61	7.24	1.52	6.80*	1.71
LET_FLU						
F	15.83	4.92	16.29	5.15	15.20	4.53
A	13.35	4.83	14.06	4.74	12.36**	4.79
CAT_FLU						
Animals	22.56	6.00	24.50	6.03	19.88***	4.83
Professions	16.21	4.94	17.70	4.76	14.12***	4.43

* p < 0.05, ** p < 0.01, *** p < 0.001, significantly different from the 60-72 years age group

Note. FA = fractional anisotropy, MD = mean diffusivity, CCG = cingulum cingulate gyrus, CHC = cingulum hippocampus, CS = corticospinal tract, FMAJ = forceps major, FMIN = forceps minor, IFOF = inferior fronto-occipital Fasciculus, SLF = superior longitudinal Fasciculus, PS = perceptual speed, EM = episodic memory, SM = semantic memory, LET_FLU = letter fluency, CAT_FLU = category fluency.

All FA and MD variables have been multiplied with 100 to make the variances more similar to those of the cognitive variables.

Table 2 shows the correlations between the latent white matter factors and the latent cognitive factors. Better performance in the PS domain was related to higher FA in FMAJ and IFOF and lower MD in CHC, CS, FMAJ, and IFOF (marginally significant, p = 0.07). When stratifying the sample according to age group, significant associations between the latent white matter factors and PS were observed in the old-old only. Expressed in terms of effect size [28], the significant associations between the white matter indicators and PS in the old-old showed medium effect sizes (rs = 0.25-0.34), whereas the corresponding (non-significant) associations in the young-old indicated small effect sizes (rs = 0.02-0.12). 

**Table 2:** Correlations of FA and MD in the seven latent white matter tracts to performance in the five cognitive domains for the total sample and for the two age groups.

	**FA**			**MD**		
	Total sample	60-72 years	78-87 years	Total sample	60-72 years	78-87 years
**PS**						
CCG	0.07	0.01	0.16	-0.10	-0.12	-0.10
CHC	0.10	0.05	0.21	-0.24*	-0.10	-0.34*
CS	0.01	-0.12	0.16	-0.16*	-0.06	-0.25*
FMAJ	0.19*	0.12	0.28*	-0.19*	-0.05	-0.31*
FMIN	0.11	0.05	0.19	-0.09	-0.08	-0.10
IFOF	0.18*	0.11	0.28*	-0.15	-0.02	-0.25*
SLF	0.01	-0.04	0.09	-0.09	-0.02	-0.14
**EM**						
CCG	0.00	0.04	-0.02	0.04	0.06	-0.02
CHC	0.08	0.16	-0.05	0.03	0.14	-0.09
CS	-0.03	0.06	-0.19	-0.04	0.03	-0.05
FMAJ	0.06	0.09	0.02	-0.08	0.06	-0.23
FMIN	0.01	0.02	-0.10	-0.00	0.03	-0.03
IFOF	0.04	0.03	0.08	-0.01	0.08	-0.09
SLF	-0.04	0.02	-0.15	0.04	0.06	0.03
**SM**						
CCG	0.00	0.04	-0.04	0.00	-0.05	0.21
CHC	-0.01	0.13	-0.29	-0.01	-0.10	-0.26
CS	-0.04	-0.02	-0.07	-0.04	-0.06	-0.02
FMAJ	0.05	0.13	-0.06	0.05	-0.08	-0.03
FMIN	-0.07	0.03	-0.14	-0.07	-0.04	0.02
IFOF	0.05	0.10	-0.04	0.05	-0.07	-0.04
SLF	-0.03	0.02	-0.09	-0.03	-0.05	0.04
**LET_FLU**						
CCG	0.01	0.01	-0.02	0.03	0.10	-0.06
CHC	0.03	-0.06	0.16	-0.07	0.07	-0.24
CS	-0.05	-0.02	-0.08	-0.07	-0.05	-0.10
FMAJ	0.09	0.08	0.10	-0.07	0.07	-0.18
FMIN	-0.07	-0.07	-0.06	0.12	0.15	0.08
IFOF	0.02	-0.07	0.12	-0.01	0.10	-0.10
SLF	-0.08	-0.08	-0.12	0.08	0.17	0.01
**CAT_FLU**						
CCG	0.08	0.12	-0.02	-0.10	-0.10	-0.11
CHC	0.12	0.24	-0.13	-0.04	-0.04	-0.04
CS	0.03	0.04	0.02	-0.05	-0.06	-0.07
FMAJ	0.04	0.16	-0.11	-0.02	-0.07	0.01
FMIN	-0.00	0.05	-0.06	0.05	-0.02	0.11
IFOF	0.14	0.20	0.01	0.00	-0.03	0.03
SLF	0.06	0.13	-0.05	-0.06	-0.07	-0.05

* p < 0.05

Note. FA = fractional anisotropy, MD = mean diffusivity, CCG = cingulum cingulate gyrus, CHC = cingulum hippocampus, CS = corticospinal tract, FMAJ = forceps major, FMIN = forceps minor, IFOF = inferior fronto-occipital Fasciculus, SLF = superior longitudinal Fasciculus, PS = perceptual speed, EM = episodic memory, SM = semantic memory, LET_FLU = letter fluency, CAT_FLU = category fluency. All analyses were adjusted for age.

Reanalyzing the data controlling for education, handedness (right-handed vs. other), or white matter lesion load (visually assessed using a modified Scheltens scale) did not alter the patterns of associations between FA, MD and PS (data not shown).

In order to examine whether the estimated correlation coefficients differed significantly between the two age groups, we performed additional analyses where we estimated the model as a multiple group model with two groups (young-old and old-old). In these analyses, we only included PS, as we had not observed any significant associations for the other cognitive factors. First, we certified measurement equivalence over the two age groups by comparing a default model with freely estimated loadings on the latent factors to a nested model that assumed the loadings to be equal across groups. The difference in chi-square fit statistics was used to compare nested models. As we did not obtain a significant difference in fit between these two models for either FA or MD (ps > 0.15), we concluded that the loadings were equivalent across age groups and the same model could be used to analyze the associations in young-old and old-old persons. Second, we compared a model where the standardized covariances between the residual terms for PS and FMAJ had been set to be equal between the young-old and the old-old to a default model where the standardized covariances were not forced to be equal for the two groups. This was repeated for all tracts where we had observed significant associations of PS to FA or MD. As the difference in model fit between the default model and the alternative model was never significant (ps > 0.10), we conclude that the size of the correlation coefficients were not significantly different between the young-old and the old-old. 

Of note is that the majority of the variance associated with the relation between white matter microstructure and cognition was shared with age. When age was entered as a covariate, the strength of the correlations between the latent tract factors and the latent cognitive factors was reduced by 50% or more (zero-order correlations not shown). 

Given that older age is associated with an increased dementia risk [29], it is possible that the strong relationships of FA and MD to PS in the old-old could be due to the presence of preclinical dementia cases in this age group. According to preliminary dementia diagnoses (DSM-IV criteria) available at follow-up, 5 persons in the young-old and 12 persons in the old-old age group developed dementia during the follow-up period (maximum = 6 years). Hence, we repeated the analyses before and after excluding persons in a preclinical dementia phase. These analyses were only performed for PS, as this was the only domain significantly associated with the white matter indicators. The results from these analyses are shown in Table 3. For FA, there was no effect of excluding the future dementia cases. However, for MD the strength of the relationships of CHC and CS to PS was attenuated and did not remain significant in the reduced sample. 

**Table 3:** Correlation coefficients of FA and MD to perceptual speed before and after exclusion of persons in a preclinical dementia phase.

	FA				MD			
	60-72 years		78-87 years		60-72 years		78-87 years	
	Before exclusion	After exclusion	Before exclusion	After exclusion	Before exclusion	After exclusion	Before exclusion	After exclusion
CCG	0.02	0.03	0.19	0.15	-0.12	-0.13	-0.15	-0.13
CHC	0.05	0.07	0.24	0.35	-0.10	-0.15	-0.38*	-0.28
CS	-0.11	-0.10	0.18	0.14	-0.06	-0.07	-0.31*	-0.27
FMAJ	0.11	0.14	0.34**	0.39**	-0.05	-0.06	-0.34*	-0.37*
FMIN	0.05	0.06	0.24	0.20	-0.08	-0.07	-0.14	-0.15
IFOF	0.11	0.10	0.30*	0.28*	-0.02	-0.03	-0.28*	-0.31*
SLF	-0.04	-0.03	0.15	0.16	-0.02	-0.04	-0.21	-0.23

* p < 0.05, **p < 0.01

Note. FA = fractional anisotropy, MD = mean diffusivity, CCG = cingulum cingulate gyrus, CHC = cingulum hippocampus, CS = corticospinal tract, FMAJ = forceps major, FMIN = forceps minor, IFOF = inferior fronto-occipital Fasciculus, SLF = superior longitudinal fasciculus. All analyses were adjusted for age.

## Discussion

We observed a significant association between white matter microstructure and perceptual speed. In exploring this association further, the link was reliable among the very old (≥78 years), but not in the 60-72 years-old subsample. These observations were made in a sample free from dementia and other neurological disorders, and remained also after excluding persons in a preclinical dementia phase.

The finding that white matter microstructure was associated with perceptual speed is in accordance with previous research on elderly samples [4-6]. Alterations in white matter integrity likely lead to less efficient communication among brain networks, and it is conceivable that this affects processing speed to a greater extent than other cognitive functions. It has been suggested that disconnection of cortical areas and functional disruption of large-scale neurocognitive networks, as a result of decline in white matter integrity, may be one underlying mechanism for age-related deficits in cognitive functioning [30,31]. In the present study, the strongest associations with perceptual speed were observed for FA and MD in FMAJ and IFOF. These are both large white matter fiber tracts connecting the occipital lobes through the corpus callosum (FMAJ) and to the frontal lobes (IFOF). Given that perceptual speed requires the involvement of multiple brain regions, associations between major association pathways and cognitive functioning is in line with the disconnection hypothesis of cognitive aging. 

Although the participants did not have any apparent motor problems (as noted by the test administrator), it cannot be ruled out that a motor component played a role in the white matter-speed association observed, given that both speeded tasks used in this study are dependent on motor abilities. For example, Vernooij et al. [6] observed a relationship of FA and MD to motor speed. That said, Sasson et al. [32] found an association between white matter integrity and processing speed also after controlling for motor function.

When stratifying the sample according to age, the associations between white matter and perceptual speed were non-significant in the young-old, but reliable in the old-old. There are several possible explanations for this pattern of results. First, larger effect sizes in the older age group may indicate that individual differences in change of white matter (i.e. decrease of FA and increase of MD) and perceptual speed (i.e. cognitive decline) are associated. If this is the case, between-person differences in white matter integrity and speed at a certain point in time will be more determined by aging-related influences, and less by initial individual differences, the older the sample is. As a result, correlations between white matter integrity and perceptual speed would be expected to increase with age [10,11,33]. It should be noted, however, that we did not find the correlation coefficients between the young-old and old-old to be significantly different. Furthermore, this reasoning is based on the typical assumption in cross-sectional research, such as absence of age-differential selection effects. Longitudinal work is needed to directly address the developmental association between white matter microstructure and cognition in aging.

Another possible explanation for why the associations with perceptual speed were only observed in the older age group is that the integrity of white matter tracts becomes more important after declining below a certain threshold. White matter integrity decreases across the adult life span, starting after age 40 [34]. However, it might be that it is only after a certain level of degeneration that white matter integrity starts to affect cognitive performance. Indeed, stronger associations between brain structure and cognitive functioning have been observed in older compared to younger age groups in previous studies [35,36].

Even though we only observed an association between indices of white matter microstructure and speed in the very old, this does not mean that there is no relationship between a well-functioning white matter network and perceptual speed at younger ages. For example, associations between white matter integrity and speeded performance have been observed also in young adults [37,38]. The reason why we did not observe a significant association in the young-old may be that we were only able to assess FA and MD in central parts of large white matter tracts due to limitations in the data. With more sensitive measures of white matter integrity (e.g. assessing white matter integrity in smaller tracts, or in tracts closer to the cerebral cortex), or cognitive functioning (e.g. reaction time measures), associations to perceptual speed may be observed also at younger ages.

Finally, the significant correlation between white matter integrity and perceptual speed in the old-old age group may partly reflect changes related to incipient dementia. Decreases in white matter integrity have been reported in persons with pre-symptomatic Alzheimer’s disease [39], amnestic mild cognitive impairment [40], and Alzheimer’s disease [40,41]. The regional distribution of decreased white matter integrity in these samples is widespread but often follows the pathology of AD, with early alterations in subregions of the medial temporal lobe [42]. To examine this issue, we repeated the analyses after excluding all persons who received a dementia diagnosis during the available follow-up period (maximum 6 years). For FA, the associations between perceptual speed and FMAJ and IFOF remained unchanged in the reduced sample. Also for MD, the strength of the associations with these tracts remained unchanged. However, excluding persons with impending dementia resulted in weaker associations of MD in CHC and CS to perceptual speed. As CHC is a tract located in the medial temporal lobe, it is possible that these correlations were partly associated with AD-related pathology.

Major strengths of the present study are that we assessed the association between white matter microstructure and cognitive functioning in a large population-based sample of elderly persons, also including the very old. This made it possible to examine the associations in different age segments in late adulthood. Further, we examined white matter-cognition links for a range of cognitive domains at the latent level, thereby reducing effects related to specific cognitive measures and at the same time removing error variance associated with a particular task. An additional advantage is that we had access to follow-up information on most subjects, and thus were able to screen the sample for incipient dementia. However, some limitations should be noted. The data collection took place between 2001 and 2004. Thus, the DTI measurements are not of modern quality. Though forming latent variables attenuated the influence of error variance, large and anisotropic voxels may have introduced partial volume effects (grey/white mixture) that could not be completely accounted for by the TBSS processing. Another limitation is that the analyses are based on cross-sectional data, which prevents us from making direct inferences about within-person changes. Longitudinal studies within this area are greatly needed.

## Supporting Information

File S1 **Supporting tables**.
Table S1, Standardized loadings on the latent factors in the combined structural equation models of fractional anisotropy (FA) and mean diffusivity (MD) for the total sample. Table S2, Correlations among the latent white matter tract factors in the combined structural equation models for fractional anisotropy (below the diagonal) and mean diffusivity (above the diagonal) for the total sample. Table S3, Correlations among the latent cognitive factors in the combined structural equation models for fractional anisotropy (below the diagonal) and mean diffusivity (above the diagonal) for the total sample.
(DOCX)
