# Supplementary material for: Associations between White Matter Microstructure and Cognitive Performance in Old and Very Old Age
Source: PLoS One. 2013 Nov 25;8(11):e81419. doi: 10.1371/journal.pone.0081419 (PMC3839877; doi:10.1371/journal.pone.0081419)
Supplement: File S1 — Supporting tables. Table S1, Standardized loadings on the latent factors in the combined structural equation models of fractional anisotropy (FA) and mean diffusivity (MD) for the total sample. Table S2, Correlations among the latent white matter tract factors in the combined structural equation models for fractional anisotropy (below the diagonal) and mean diffusivity (above the diagonal) for the total sample. Table S3, Correlations among the latent cognitive factors in the combined structural equation models for fractional anisotropy (below the diagonal) and mean diffusivity (above the diagonal) for the total sample. (DOCX) [file pone.0081419.s001.docx]

**Table S1.** Standardized loadings on the latent factors in the combined structural equation models of fractional anisotropy (FA) and mean diffusivity (MD) for the total sample.

|  | FA | MD |
| --- | --- | --- |
| CCG_L | 0.88 | 0.85 |
| CCG_R | 0.93 | 0.91 |
| CHC_L | 0.71 | 0.77 |
| CHC_R | 0.72 | 0.85 |
| CS_L | 0.95 | 0.97 |
| CS_R | 0.95 | 0.93 |
| FMAJ_L | 0.92 | 0.86 |
| FMAJ_R | 0.89 | 0.90 |
| FMIN_L | 0.95 | 0.94 |
| FMIN_R | 0.95 | 0.95 |
| IFOF_L | 0.94 | 0.92 |
| IFOF_R | 0.94 | 0.93 |
| SLF_L | 0.94 | 0.97 |
| SLF_R | 0.91 | 0.93 |
| PS |  |  |
| Digit cancellation | 0.63 | 0.63 |
| Pattern comparison | 0.87 | 0.88 |
| EM |  |  |
| Word recall | 0.94 | 0.91 |
| Word recognition | 0.48 | 0.50 |
| SM |  |  |
| Vocabulary | 0.99 | 0.93 |
| General knowledge | 0.39 | 0.41 |
| LET_FLU |  |  |
| F | 0.82 | 0.82 |
| A | 0.89 | 0.88 |
| CAT_FLU |  |  |
| Animals | 0.84 | 0.82 |
| Professions | 0.73 | 0.75 |

*Note.* FA = fractional anisotropy, MD = mean diffusivity, CCG = cingulum cingulate gyrus, CHC = cingulum hippocampus, CS = corticospinal tract, FMAJ = forceps major, FMIN = forceps minor, IFOF = inferior fronto-occipital fasciculus, SLF = superior longitudinal fasciculus, PS = perceptual speed, EM = episodic memory, SM = semantic memory, LET_FLU = letter fluency, CAT_FLU = category fluency.

**Table S2.** Correlations among the latent white matter tract factors in the combined structural equation models for fractional anisotropy (below the diagonal) and mean diffusivity (above the diagonal) for the total sample.

| Factor | CCG | CHC | CS | FMAJ | FMIN | IFOF | SLF |
| --- | --- | --- | --- | --- | --- | --- | --- |
| CCG | - | 0.70* | 0.74* | 0.77* | 0.84* | 0.80* | 0.81* |
| CHC | 0.65* | - | 0.63* | 0.86* | 0.67* | 0.76* | 0.58* |
| CS | 0.75* | 0.48* | - | 0.81* | 0.78* | 0.86* | 0.87* |
| FMAJ | 0.80* | 0.47* | 0.62* | - | 0.81* | 0.95* | 0.81* |
| FMIN | 0.88* | 0.54* | 0.72* | 0.76* | - | 0.86* | 0.85* |
| IFOF | 0.79* | 0.65* | 0.68* | 0.76* | 0.82* | - | 0.92* |
| SLF | 0.84* | 0.59* | 0.83* | 0.70* | 0.78* | 0.80* | - |

*Note.* FA = fractional anisotropy, MD = mean diffusivity, CCG = cingulum cingulate gyrus, CHC = cingulum hippocampus, CS = corticospinal tract, FMAJ = forceps major, FMIN = forceps minor, IFOF = inferior fronto-occipital fasciculus, SLF = superior longitudinal fasciculus.

* significant correlation at p < 0.001.

**Table S3.** Correlations among the latent cognitive factors in the combined structural equation models for fractional anisotropy (below the diagonal) and mean diffusivity (above the diagonal) for the total sample.

| Factor | PS | EM | SM | LET_FLU | CAT_FLU |
| --- | --- | --- | --- | --- | --- |
| PS | - | 0.42* | 0.49* | 0.54* | 0.70* |
| EM | 0.42* | - | 0.25* | 0.32* | 0.50* |
| SM | 0.46* | 0.23* | - | 0.47* | 0.47* |
| LET_FLU | 0.54* | 0.32* | 0.44* | - | 0.61* |
| CAT_FLU | 0.70* | 0.49* | 0.43* | 0.60* | - |

*Note.* PS = perceptual speed, EM = episodic memory, SM = semantic memory, LET_FLU = letter fluency, CAT_FLU = category fluency.

* significant correlation at p < 0.001.
